# Carotid intima-media thickness in individuals with and without type 2 diabetes: a reproducibility study

**DOI:** 10.1186/1475-2840-9-40

**Published:** 2010-08-20

**Authors:** Louise Lundby-Christensen, Thomas P Almdal, Bendix Carstensen, Lise Tarnow, Niels Wiinberg

**Affiliations:** 1 Steno Diabetes Center, Niels Steensens vej 2-4, 2820 Gentofte, Denmark; 2 Frederiksberg Hospital, dept. of clinical physiology and nuclear medicine, Nordre Fasanvej 57-59, 2000 Frederiksberg, Denmark

## Abstract

**Background:**

The use of carotid intima-media thickness (carotid IMT) as a surrogate marker of cardiovascular disease is increasing and the method has now also been applied in several trials investigating patients with type 2 diabetes (T2D). Even though knowledge about methodology is of highest importance in order to make accurate power calculations and analyses of results, no reproducibility studies have been performed in this group of patients. The aim of this study was to quantify the variability of the measurement of carotid IMT in individuals with and without T2D.

**Methods:**

We used B-mode ultrasound and a computerized software programme (MIA vascular tools) for analysis of carotid IMT. Measurement of carotid IMT in the far wall of the common carotid artery (CCA) was done for 30 patients with T2D and 30 persons without T2D. The examinations were done by two different sonographers and two different readers on two separate days in order to quantify sonographer-, reader-, and day-to-day variability.

**Results:**

Comparisons of measurement of carotid IMT in CCA between sonographers (sonographer variability) resulted in limits of agreement (LoA) from -0.18 to 0.13 mm for patients with T2D and -0.12 to 0.10 mm for persons without T2D. This means, that a second scanning of the same person with 95% probability would be within this interval of the first scanning. Comparisons between readers assessing the same scanning (reader variability) resulted in LoA from -0.05 to 0.07 mm and -0.04 to 0.05 mm respectively. LoA of the day-to-day variability was -0.13 to 0.18 mm and -0.09 to 0.18 mm respectively. This corresponds to coefficients of variations (CV) of the sonographer- and day-to-day variability of 10% in patients with T2D and 8% in persons without T2D. The CV of the reader variability was 4% and 3% respectively.

**Conclusion:**

Measurement of carotid IMT in the CCA can be determined with good and comparable reproducibility in both patients with T2D and persons without T2D. These findings support the use of carotid IMT in clinical trials with T2D patients and suggest that the numbers of patients needed to detect a given difference will be the same whether the patients have T2D or not.

## Background

Determination of carotid intima-media thickness (carotid IMT) is a generally accepted research method for detection and quantification of subclinical cardiovascular disease (CVD). It is based on the combined thickness of the tunica intima and tunica media of the carotid artery wall and is measured by B-mode ultrasound. A number of epidemiological studies reports that increased carotid IMT is a good predictor of future CVD such as myocardial infarction, stroke and death from CVD. Furthermore carotid IMT correlates well with clinically established CVD and the Framingham Score[1-5]. Based on the existing evidence from trials with statins, carotid IMT fulfils the three generally accepted requirements for a surrogate marker[6]. In addition, the measurement is non-invasive, inexpensive, readily applicable and carries virtually no risk for the patient. Therefore, carotid IMT has now become an established surrogate marker of CVD in clinical trials to evaluate the efficacy of interventions with statins, antihypertensives, aspirin and antidiabetic medications [7-12]. The primary advantages of using a surrogate marker instead of hard outcome measures such as myocardial infarction, stroke and death are smaller study populations, shorter study duration and thereby reduced financial costs.

In general, knowledge about research methodology is of highest importance and as stated by Fleiss in 1986 "The most elegant design of a clinical study will not overcome the damage caused by unreliable or imprecise measurements"[13]. This applies obviously also to measurement of carotid IMT. The technique, by which carotid IMT is determined, consists of two steps: The first step is the "scanning procedure" i.e. ultrasound scanning of the carotid artery, with storage of pictures/dynamic sequences and the second step is the "reading procedure" i.e. the following measurement of carotid IMT using a specialized software technique. Even though the variability of the technique like this is influenced by both the scanning procedure and by the following reading procedure, many reproducibility studies have only taken the variability of the reading procedure into account which does not reflect the total variability [14-16]. Furthermore, no methodological studies have explicitly evaluated the reproducibility in patients with type 2 diabetes (T2D) even though the method has now also been increasingly applied in clinical trials with diabetes patients [10-12,17-19].

Patients with T2D have a two to fourfold increased incidence of CVD compared to persons without diabetes [20,21], which is also reflected by an increased carotid IMT in T2D patients [22-24]. Earlier studies have suggested that the variability of the measurement of carotid IMT increases with increasing carotid IMT[25]. Hence, it could be argued that the variability is increased in T2D patients compared to persons without T2D, but this has not been assessed systematically.

The purpose of this study was to quantify the variability between sonographers, between readers and between days of the measurement of carotid IMT in the far wall of the common carotid artery (CCA) in patients with T2D and persons without T2D.

## Methods

### Patients

Sixty persons participated in this study; 30 patients with T2D and 30 persons without T2D.

### Visits

All participants paid two visits within two weeks (visit A and B) at Steno Diabetes Center, Copenhagen, Denmark. At each visit patients were scanned by two different sonographers (sonographer X and Y), to enable assessment of the day-to-day variability and the sonographer variability (table 1).

**Table 1:** Scanning and reading procedure for every participant for the measurement of mean carotid IMT.

Visit	Sonographer	Reader	Replicate
A	X	x	1
		x	2
		y	1
		y	2
	Y	x	1
		x	2
		y	1
		y	2
B	X	x	1
		x	2
		y	1
		y	2
	Y	x	1
		x	2
		y	1
		y	2

Every participant performed two visits (A and B). At every visit they were scanned twice by each of the sonographers X and Y, and every scanning was read twice (replicate 1 and 2) by each of the two readers (x and y).

### Scanning procedure

After 10 minutes of rest in a supine position blood pressure was measured at the left upper arm whereupon the scanning was performed using a *General Healthcare (GE) logic 9 *with a 9 linear (8 MHz) or a 12 linear (12 MHz) probe. The choice of probe depended on the most appropriate probe to the person; however, the same probe with the same frequency was used for the same person at every scanning. All examinations were carried out in a dark, quiet and temperature-controlled room.

With the head in a slightly bended position towards the opposite site of the one being scanned the ultrasound transducer was placed in an angle of 90° of the vessel wall (to obtain parallel ecco lines of the intima and media in both near and far wall). First a rough cross sectional scanning was made from the proximal part of common carotid artery (CCA) throughout the bifurcation to the internal carotid artery and the external carotid artery to localise possible plaques or stenoses. The transducer was turned and a longitudinal scanning was made in the CCA with storage of the dynamic sequence for the following reading procedure to measure carotid IMT. The depth of the scan was adjusted and the transmit focus zone set at the optimal level to show the best possible image of the far wall of the CCA. A segment of the artery was magnified using a resolution box and the grey scale image adjusted to identify a distinct lumen-intima and media-adventitia interface of the artery wall. Then, 5 seconds of the ultrasound image was digitally recorded on a computer for later measurements of the carotid IMT. This procedure was made first on the right CCA and afterwards on the left CCA.

### Reading procedure

For the border detection and calculation of the carotid IMT, we used specialized software (vascular tools 5, Medical Imaging Applications, Iowa, USA). First, the region of interest was defined as a segment of the CCA far wall devoid of focal plaques and spanning 5-10 mm with a centre 10 mm proximal to the bulb. In this region (= reading frame) the software identifies the lumen/intima and the media/adventitia borders and calculates the distance in between, i.e. the carotid IMT. The resolution of the ultrasound picture is 10 pixels/mm. The frequency of the picture is 15 pictures per second during five seconds. The final average carotid IMT on a 10 mm segment of the vessel wall is thus based on approximately 600 automated calculations during the 5 seconds. IMT was finally calculated as the mean IMT of the left and right CCA.

### Data

For each patient we obtained four scanning results, two from each of the two visits (visit A and B, table 1).

Each of these were read twice by each of the two readers (reader X and y) to measure inter- and intra-reader variability. The readers (x,y) were the same persons as the sonographers (X,Y). For each reading, the mean CCA of the left and right side were summed and averaged to yield the average mean carotid IMT resulting in a total of 16 values of average mean carotid IMT from each participant, classified by visit, sonographer, reader and replicate of reading (table 1). Replicates were considered exchangeable within each combination of visit, sonographer and reader.

### Statistical analysis

**Figure 1:**
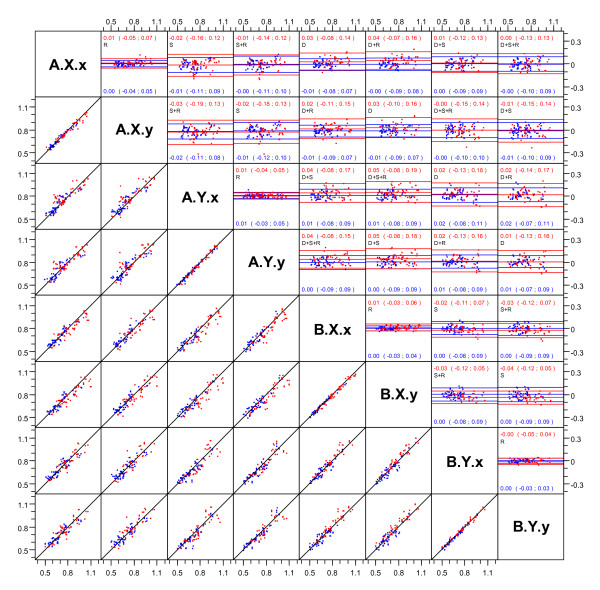
**Scatter and Bland-Altman plots of the pairwise comparisons of carotid IMT**. Measurements of carotid IMT - average of left and right side measurements on the absolute scale (mm). Comparisons across visits (A/B), sonographers (X/Y) and readers (x/y). Replicate measurements are randomly permuted within each combination of these three factors. Points and lines in red are for persons with T2D, in blue for persons without T2D. The panels above the diagonal are Bland-Altman plots of the differences of the pairwise comparisons of carotid IMT. Plots below the diagonal are just the corresponding scatter plots. Limits of agreement are given in brackets (), red for T2D and blue for persons without T2D. Visits: A and B, sonographers: X and Y, readers: X and y. Symbols in plot: S = sonographer variability, R = reader variability, D = day-to-day variability, S + R = combined sonographer and reader variability, S + D = combined sonographer and day-to-day variability, R + D = combined reader and day-to-day variability, S + R + D = combined sonographer, reader and day-to-day variability.

We computed limits of agreement (LoA) and made Bland-Altman plots between all pairs of the 8 combinations of visit, sonographer and reader (figure 1). Replicates were randomly permuted for this comparison. These analyses were performed separately for measurements on patients with T2D and persons without T2D.

Here we report the uncertainty of the measurements as standard deviation of the mean absolute difference of the pairwise comparisons (SD (diff)), LoA on the absolute scale (mm) as well as coefficient of variation (CV) calculated as the SD of the differences of the log-transformed values. The LoA is the 95% prediction interval for the difference between results from two different combinations of visit, sonographer and reader, *i.e*. there is a 95% probability that the difference between two measurements will be in this interval. Depending on which combinations of visit, sonographer and reader are compared, different components of the variations are included; this is indicated in the Bland-Altman plots too

The study complied with the Declaration of Helsinki and was approved by the Regional Ethical Committee (region of Copenhagen journal number H-A-2007-0126).

## Results

Of the 60 participants, two were excluded as the values of carotid IMT could not be obtained because of widespread atherosclerosis/plaques in the CCA. They (a man and a woman) had both had T2D for more than 15 years and clinically established CVD. Hence, 58 participants (28 patients with T2D and 30 persons without T2D) as characterised in table 2 were included in the analysis.

**Table 2:** Characteristics of participants (%/mean ± SD)

	Patients with T2D	Persons without T2D
n	28	30
Age (years)	63.4 ± 7.8	54.4 ± 10.3
Men (%)	68	47
Diabetes duration (years)	16 ± 8	0
Smokers (%)	21	10
BMI (kg/m2)	32.7 ± 4.5	26.4 ± 3.6
Microalbuminuria (%)	50	0
Prevalent CVD (%)	21	0
Total cholesterol (mmol/l)	3.76 ± 0.71	5.10 ± 1.06
HDL cholesterol (mmol/l)	1.33 ± 0.39	1.55 ± 0.33
LDL cholesterol (mmol/l)	1.76 ± 0.57	2.94 ± 0.93
Triglycerides (mmol/l)	1.45 ± 0.55	1.33 ± 0.57
Treatment with statins (%)	96	10
HbA1c (%)	7.75 ± 1.04	5.52 ± 0.22
Therapeutic methods for T2D: Diet only/OAD/insulin/OAD+insulin (%)	11/14/50/25	
Systolic blood pressure (mmHg)	132 ± 19	126 ± 13
Diastolic blood pressure (mmHg)	77 ± 9	77 ± 8
Antihypertensive treatment (%)	96	7
Therapeutic method for hypertension: ACEi/ARB/β-blockers/diuretics/CCB (%)	39/54/11/68/32	0/3/0/3/0
Mean carotid IMT (mm)	0.800 ± 0.131	0.679 ± 0.105

OAD: Oral antidiabetics, ACEi: angiotensin-converting enzyme inhibitor, ARB: angiotensin II receptor blocker, CCB: calcium channel blocker

Mean carotid IMT in the far wall CCA in patients with T2D was 0.800 ± 0.131 mm (mean ± SD). 50% of the patients with T2D were diagnosed with microalbuminuria (albumin-to-creatinin ratio 30-300 mg/g in ≥ two of three consecutive urine samples). Significantly higher values of carotid IMT were found in the 14 patients with microalbuminuria (0.867 mm ± 0.122) as compared to the 14 patients with normoalbuminuria (0.733 mm ± 0.106, p < 0.005) despite no difference in age (62.1 years ± 7.1 vs. 64.7 ± 8.3, p = 0.4) or duration of T2D (15.8 years ± 8.4 vs. 16.9 ± 7.3, p = 0.7). In persons without T2D mean carotid IMT was 0.679 ± 0.105 mm. Nearly all patients with T2D were treated with simvastatin 40 mg (96%) whereas this was the case for only a very little proportion of the healthy persons (10%). 96% of the patients with T2D received antihypertensive treatment in terms of angiotensin converting enzyme inhibitors (ACEi, 39%), angiotensin II receptor blockers (ARB, 54%), β-blockers (11%), diuretics (68%) and/or calcium channel blockers (CCB, 32%). Only two persons without T2D received antihypertensive treatment (ARB and diuretic respectively). Mean systolic blood pressure among patients with T2D was 132 ± 19 mmHg compared to 126 ± 13 mmHg among persons without T2D. Mean diastolic blood pressure was 77 ± 9 mmHg and 77 ± 8 mmHg respectively.

Glucose lowering therapies were given as follows: 11% received diet only, 25% received oral antidiabetics (OAD's, i.e. metformin ± sulfonylurea) only, 64% received insulin (either long-acting (32%), rapid-acting (11%) and/or biphasic insulin (32%).

The scatter plots and Bland-Altman plots with LoA indicated are shown in Figure 1, in blue for healthy persons and red for patients with T2D. Overall, there were no systematic difference between any of the combinations of day, sonographer and reader.

### Variability of measurement of carotid IMT in patients with T2D

When different scanning results were assessed (*sonographer variability*), the LoA were -0.18 to 0.13 mm. This means, that a second scanning of the same person with 95% probability would be within this interval of the first scanning. (Figure 1; LoA for T2D patients indicated by red numbers and lines, sonographer variability marked by "s"). The SD (diff) was 0.078 mm. When the same scanning's were assessed by different readers (*reader variability*), the limits of agreement were -0.05 to 0.07 mm and the SD (diff) was 0.030 mm. When comparing scanning's made by the same sonographer and read by the same reader but assessed on two different days (*day-to-day variability*) the limits of agreement were -0.13 to 0.18 mm and the SD (diff) was 0.075 mm (Figure 1).

### Variability of measurement of carotid IMT in persons without T2D

When different scanning results were assessed (*sonographer variability*), the limits of agreement were -0.12 to 0.10 mm and the SD (diff) was 0.055 mm (figure 1). When the same scanning's were assessed by different readers (*reader variability*), the limits of agreement were -0.04 to 0.05 mm and the SD (diff) was 0.023 mm. When comparing scanning's made by the same sonographer and read by the same reader but assessed on two different days (*day-to-day variability*) the limits of agreement were -0.09 to 0.18 mm and the SD (diff) was 0.050 mm.

The coefficient of variation (CV) as measured by the SD of the log-transformed values was 10% between sonographers and between days (10% for patients with T2D and 8% for persons without T2D) and 4% between readers (4% for patients with T2D and 3% for persons without T2D).

## Discussion

The use of carotid IMT has been increasingly used in trials investigating patients with T2D. The existing knowledge about reproducibility of this method is based on very heterogeneously populations including either no or relatively few patients with T2D[15,16]. With regard to future clinical diabetes research further knowledge about reproducibility of this method is required. In this methodological study we have explicitly evaluated the reproducibility of the method in patients with T2D and persons without T2D and we have accurately pointed out the main sources and sizes of variability. The study shows that carotid IMT can be determined with good and comparable reproducibility in both patients with T2D and healthy persons

The patients with T2D participating in this study had a mean diabetes duration of 16 ± 8 years, 50% had microalbuminuria, 21% had clinical established CVD and the mean age was 63,4 ± 7,8 years which confirms that we deal with a group of patients with advanced disease. In contrast, the persons without T2D were younger (54.4 ± 10,3 years) and had no clinical established CVD. The main source of variability in the measurement of carotid IMT was found in the scanning procedure with a CV of 10% and 8% for patients with T2D and persons without T2D respectively. This variability corresponds well with the sonographer variability reported in other studies performed in persons without T2D or in some cases appears to be of even smaller magnitude [25-27]. The day-to-day variability was identical to the sonographer variability for both patients with T2D and persons without T2D (CV of 10% and 8% respectively) which likewise is in agreement with earlier studies in normoglycaemic individuals[28]. Only a minor source of variability was found in the reading procedure (CV of 4% and 3% respectively) as reported earlier in persons without T2D[16]. So even though the patients with T2D in this study had an advanced disease, were approximately 9 years older, had higher BMI, higher systolic blood pressure and a higher proportion of males, the variability of the measurement of carotid IMT were nearly identical to the persons without T2D. It should be noted, however, that this study only used two sonographers, so it could be argued, that more sonographers would increase the sonographer variability. However, as the variability between scanning results by the same sonographer from different days (day-to-day variability) is of the same order of magnitude as the variability between different sonographers on the same day (sonographer variability), it is unlikely that this would be the case.

In addition, we found a significant increase in carotid IMT independent of age and duration of diabetes in the T2D patients with microalbuminuria compared to the patients with normoalbuminuria. Accordingly, earlier studies have found a significant association between carotid IMT and diabetic nephropathy measured as either microalbuminuria (elevated ACR) and/or decreased estimated glomerular filtration rate (eGFR)[29-31].

## Conclusions

In conclusion, this study demonstrates that measurement of carotid IMT can be performed in patients with T2D without markedly increasing the variability of the measurement as compared to persons without T2D. The major source of variability is found in the scanning procedure, which has nearly identical variability as the day-to-day variability, whereas very little variability is found in the following reading procedure.

Thus, given the present results studies using carotid IMT as outcome will be subject to nearly the same variability related to methodology whether they are performed in patients with T2D or in persons without. These results justify the use of carotid IMT in trials with T2D patients and suggest that the numbers of patients needed to detect a given difference in a clinical trial will be the same whether the patients have T2D or not.

## Competing interests

The authors declare that they have no competing interests.

## Authors' contributions

LLC, TPA, LT and NW conceived of the study and participated in its design, coordination and helped to draft the manuscript. BXC participated in the design, helped to draft the manuscript and performed the statistical analyses. All authors read and approved the final manuscript.
